# Comparative Analysis of the Effectiveness of Performing Advanced Resuscitation Procedures Undertaken by Two- and Three- Person Basic Medical Rescue Teams in Adults under Simulated Conditions

**DOI:** 10.3390/ijerph18094834

**Published:** 2021-04-30

**Authors:** Kamil Krzyżanowski, Daniel Ślęzak, Sebastian Dąbrowski, Przemysław Żuratyński, Wioletta Mędrzycka-Dąbrowska, Paulina Buca, Paweł Jastrzębski, Marlena Robakowska

**Affiliations:** 1 Department of Medical Rescue, Faculty of Health Sciences with the Institute of Maritime and Tropical Medicine, Medical University of Gdańsk, Dębinki 7, 80-211 Gdańsk, Poland; kamil.krzyzanowski@gumed.edu.pl (K.K.); sebastian.dabrowski@gumed.edu.pl (S.D.); przemyslaw.zuratynski@gumed.edu.pl (P.Ż.); 2 Department of Anaesthesiology and Intensive Care Nursing, Faculty of Health Sciences with the Institute of Maritime and Tropical Medicine, Institute of Nursing and Midwifery, Medical University of Gdańsk, Dębinki 7, 80-211 Gdańsk, Poland; wioletta.medrzycka-dabrowska@gumed.edu.pl; 3 Division of Hyperbaric Medicine & Maritime Rescue—National Centre for Hyperbaric Medicine, Faculty of Health Sciences with the Institute of Maritime and Tropical Medicine, Institute of Maritime and Tropical Medicine, Medical University of Gdańsk, Powstania Styczniowego 9b, 81-519 Gdynia, Poland; paulina.buca@gumed.edu.pl; 4 Departament of Emergency Medicine, Faculty of Health Science, University of Warmia and Mazury, Żołnierska 18, 10-561 Olsztyn, Poland; pawel.jastrzebski@uwm.edu.pl; 5 Division of Public Health and Social Medicine, Faculty of Health Sciences with the Institute of Maritime and Tropical Medicine, Medical University of Gdańsk, Tuwima 15, 80-210 Gdańsk, Poland; marlena.robakowska@gumed.edu.pl

**Keywords:** emergency medical teams, simulation, advanced rescue operations, algorithm

## Abstract

(1) Objective: Paramedics as a profession are a pillar of the State Medical Rescue system. The basic difference between a specialist and a basic team is the composition of members. The aim of the study was to benchmark the effectiveness of performing advanced resuscitation procedures undertaken by two- and three-person basic emergency medical teams in adults under simulated conditions. (2) Design: The research was observational. 200 two- and three-people basic emergency medical teams were analyzed during advanced resuscitation procedures, ALS (Advanced Life Support) in adults under simulated conditions. (3) Method: The study was carried out among professionally active and certified paramedics. It lasted over two years. The study took place under simulated conditions using prepared scenarios. (4) Results: In total, 463 people took part in the study. The analysis of the survey results indicates that the efficiency of three-person teams is superior to the activities performed by two-person teams. Three-person teams were quicker to perform rescue actions than two-person teams. The two-person teams were much quicker to assess the condition of victims than the three-person teams. The three-person teams were more likely to check an open airway. The three-person teams were more efficient in assessing the heart rhythm and current condition of victims. It was demonstrated that three-person teams were more effective during electrotherapy. The analysis demonstrated that three-person teams were significantly faster and more efficient in chest compressions. Three-person teams were less likely to use emergency airway techniques than two-person teams. The results indicate that three-person teams administered the first dose of adrenaline significantly faster than two-person teams. For the “call for help”, the three-person teams were found to be more effective. (5) Conclusion: Paramedics in three-person teams work more effectively, make a proper assessment of heart rhythm and monitor when taking advanced actions. The quality of ventilation and BLS in both groups studied is insufficient. Numerous errors have been observed in two-person teams during pharmacotherapy.

## 1. Introduction

Paramedics as a profession are a pillar of the Polish Medical Rescue (PMR) system. The PMR system is an important component of the health care system established to act in the field of saving lives and human health in emergencies. It was created to carry out the tasks of the state consisting in providing assistance to any person in case of sudden health risk [1]. A type of health services specific to medical rescuers, which they are entitled to provide are medical rescue operations. They are healthcare services within the meaning of the provisions on publicly funded healthcare services, granted by the PRM unit in the form of a medical rescue team of both specialist nature, as well as basic, in non-hospital conditions, to save a person in a state of sudden health risk. The basic difference between a specialist and a basic team is the composition. The means of transport is the same and the equipment in both cases is similar [2]. The Specialist Rescue Team (ZRMS) consists of at least three people whose education allows them to undertake medical rescue operations. The head of the unit is a system doctor, which has extended eligibility (for example will administer more medications or perform endotracheal intubation in all of the cases) and the members are medical rescuers and/or system nurses [3]. In the case of the Basic Medical Rescue Team (ZRMP) there is no doctor among the staff, thus the members have limited list of pharmaceuticals to 47 items and compared to doctor, they could perform endotracheal intubation merely in cardiac arrest cases. Interventions are undertaken by rescuers and/or system nurses, and one of these people acts as a leader. Current legal acts only specify that a rescue team must consist of minimum two people with authorization to conduct medical rescue operations [1,2]. As personal requirements are not precisely defined, there are entities where ZRMP consists of three paramedics and/or nurses. From the work ergonomics’ perspective, it seems that increasing the staff by one paramedic will positively affect work efficiency and contribute to reducing the psychophysical burden in the crew. This may be particularly important in the case of cardiopulmonary resuscitation, when the speed and quality of the introduced procedures affect the final result and condition for the return of spontaneous circulation, ROSC [4].

### Aim

The aim of the study was to benchmark the effectiveness of performing advanced resuscitation procedures undertaken by two- and three-person basic emergency medical teams in adults under simulated conditions.

## 2. Methodology

### 2.1. Design

The research was observational. 200 two- and three-person basic emergency medical teams were analyzed during advanced resuscitation procedures, ALS (Advanced Life Support) in adults under simulated conditions. The groups were created randomly. Ongoing research was not regular training. The teams were assessed according to ERC guidelines.

### 2.2. Participants

The study was conducted among professionally active qualified medical rescuers and was conducted from 2016 to 2019. In total, 463 paramedics took part in the study. Participants were divided into 100 two-person teams and 100 three-person teams at random. The sole criterium that the participants had to meet was medical paramedic diploma. Under current law, every paramedic in Poland has the same qualifications. The seniority and type of completed school do not affect the scope of the paramedic’s rights. All of the participants approved of involvement in data.

### 2.3. Procedure

Among all teams, 100 scenarios for cardiac arrest with defibrillation and non-defibrillation rhythms have been prepared. A checklist (Supplementary S1: Simulation Evaluation Checklist) has been prepared for each scenario, which took into account the correctness and duration of individual elements of the ALS algorithm. The following criteria were verified: assessment of basic life functions based on a ABC scheme (A—Airways, B—Breathing, C—Circulation), time to call for help, defibrillation technique (time to get the rhythm record—quick look; selection of energy; time of first defibrillation, time between defibrillation; comparsion of energy; use of multifunctional electrodes, arrangement of electrodes; rhythm analysis in two minutes sequences; use of the gel before defibrillation), quality of chest compression and ventilation, oxygen therapy, airway management technique, knowledge of current algorithms in cardiac arrest, pharmacotherapy and drug dosage.

ALS simulator (model AmbuMan Advanced) with computer analysis enabling constant quality control of operations and equipment compatible with the equipment of basic emergency medical teams, were used for the study (quality of chest compression, ventilation volume and time of defibrillation). The laboratory where the simulation was conducted was closed in order to prevent accidental individuals from interrupting the task. Lighting and thermal conditions were similar in each case. Before starting the task, all participants drew the script from the group of defibrillation (ventricular fibrillation, ventricular tachycardia) and non-defibrillation rhythms (asystole, pulseless electrical activity). They were given time to get acquainted with task assumptions. The scenarios differed in the description of the surroundings (bus stop, staircase, pavement, etc.), however the elements regarding the condition of the victim were unchanging (state of consciousness, quality of breath and signs of circulation). None of the prepared scenarios covered the subject of specific conditions or threats arising from the surrounding environment. They were based only on a universal algorithm for treating adult cardiac arrest. Each team, before starting the task, had time to prepare and arrange, at their sole discretion, available equipment in bags and rescue backpack. The participants had two options of airway management, first of all it was alternative method (LT—laryngeal tube, Igel—type of laryngeal mask airway), secondly was endotracheal intubation. They were also provided with access to several defibrillator models. Equipment selection, preparation method and assignment of functions in the team were not imposed. Rescuers were not informed which elements were assessed and what the purpose of the study was. Real performance of all medical procedures was recommended and rescuers were informed about its need several times before starting the simulations. The participating team members were given time to familiarize themselves with the equipment, allowed to choose and freely arrange the equipment in their medical bags/backpacks before proceeding with the scenario. The rescuers were not given information about the condition of the casualty and expected cardiac arrest rhythms. Their task was to assess the condition of the casualty and choose the optimal path of action. The scenario was discontinued after 10 min from the moment the medical actions were undertaken, regardless of the stage of the procedures. In conclusion all of the participants received outcomes of the data. Nevertheless, the debriefing held after all simulations have completed.

### 2.4. Statistical Analysis

Statistical analyzes were carried out to answer the research questions posed using the IBM SPSS Statistics 24 package (IBM^®^ SPSS^®^ Statistics; New York, NY, USA). It was used to analyze basic descriptive statistics, as well as Student’s *t*-test for independent trials, the Mann–Whitney U test and chi-square tests. In all calculations, *p* < 0.05 was assumed as the level of significance.

## 3. Results

### 3.1. Fieldwork

The study was conducted on a group of qualified, professionally active paramedics. In total, 463 people took part in the study. The age range of participants was between 26 and 48 years; 22.5% (104 people) of the respondents were women, the remaining 77.5% (359 people) were men.

### 3.2. Analysis of the Correctness and Speed of Activities Two- and Three-Person Basic Emergency Medical Teams during ALS

The first factor assessed was the time to initiate emergency procedures. The three-person teams started the procedures in shorter time compared to two-person teams Two-person teams used less time to assess the condition of the injured person compared to three-person teams. The difference between the values is statistically significant (*p* = 0.002). When analyzing the results for checking the patency of the airway (A), we note that the three-person teams performed this procedure more often than the two-person teams. When analyzing the quantitative measurements of the study, we found that the result of the Student’s *t*-test for independent samples is statistically significant for measurement B, C, and the combined measurement. Higher scores were obtained by teams of 3 versus teams of 2 (*p* = 0.032). When measured together, the three-person teams achieved a mean score close to 10 s.

The time to obtain the rhythm recording by the three-person teams was also shorter compared to the two-person teams (*p* = 0.001). Of note, there was a statistically significant (*p* = 0.001) shorter time for three-person teams to administer electrotherapy compared to two-person teams. In-depth analysis showed that three-person teams were more likely to use gel compared with two-person teams, significantly less likely to delay defibrillation, and more likely to perform rhythm analysis and discharge. A chi-square test was used to verify whether there was a relationship between team groups and the following measures: use of gel for rhythm and defibrillation assessment, defibrillation energy grading, safety, chest compressions when charging the defibrillator, defibrillation delay, rhythm analysis every 2 min, and discharge every 2 min. Statistically significant relationships were obtained for gel use, defibrillation delay, analysis every 2 min, and discharge every 2 min.

When presenting the time to undertake effective chest compressions, we note that three-person teams performed this activity in less time compared to two-person teams (*p* = 0.007). The correct rate of first chest com-pressions in both groups exceeded the recommended 120 compressions/min. Shorter intervals of first chest compressions are noticed in the activities of three-person teams. The difference was statistically significant at *p* = 0.001. Additionally, a greater depth value was obtained by the three-person teams, closest to the desired value of 50–60 mm (*p* = 0.009).

Analysis of ventilation variables showed no differences between 2- and three-person teams in terms of ventilation volume. Notably, none of the groups compared achieved volumes in the desired range of 500–600 mL. Statistically significant differences were obtained in terms of airway clearance with supraglottic methods. Lower scores were obtained by three-person teams. Statistically significant correlations were also obtained for LT and I-gel variables (*p* = 0.001). It appears that three-person teams use I-gel techniques less frequently and LT techniques more frequently than two-person teams, with both groups of teams using I-gel more frequently than LT.

Conducted pharmacotherapy, and in particular the measurement of the time of administration of the first dose of Adrenaline shows that three-person teams are statistically significantly faster than two-person teams (*p* = 0.001). The three-person teams were significantly more likely to use appropriate drug dosing compared to the two-person teams. In contrast, there was no significant relationship between appropriate dosage and team groups. The chi-square test yielded a statistically significant relationship between groups and dosing every 3–5 min.

When presenting the time of calling additional services to the scene, three-person teams performed this in a shorter period of time than two-person teams (*p* = 0.001).

Analysis of the results of the study indicates that there is a significant statistical advantage in the efficiency of three-person teams compared to activities conducted at the two-person team level. A detailed summary of the comparison of the correctness and speed of actions of 2- and three-person teams is presented in Table 1.

## 4. Discussion

Analyzing the literature, does not display any sources addressing the issue of verifying the effectiveness of work within rescue teams depending on the composition of units. The authors of numerous publications enumerate and analyze elements which may have a potential impact on obtaining ROSC, but do not discuss the impact of the number of paramedics on the quality of operations. The discussion on the impact of the numerical composition of the team on the possibility of obtaining ROSC can be based on the analysis and comparison of efficiency both units in individual components ultimately forming the entire resuscitation and the opinion of specialists regarding their impact on survival outcomes after cardiac arrest.

The authors of the European Resuscitation Council Guidelines clearly indicate the need to call for help as a matter of urgency in the event of cardiac arrest [5]. It is evident that the number of people, in addition to qualifications and experience, might be crucial here. During resuscitation or after obtaining ROSC, the competence of rescuers is not sufficient to provide further specialist care for victims. The use of muscle relaxants, pressure amines and anesthetics exceeds the authority of intermediate medical staff.

Evacuation of the victim is another factor indicating that the number of paramedics can have a positive impact on the pace of work. The weight of the victim, room dimensions, the number and weight of equipment used to maintain life functions mean that preparation for transport consumes a lot of energy and takes a great amount of time. The study showed that out of both groups of teams, three-person units faster asked other teams for support and thus—they received the expected help faster. The stance of the author of the study and specialists from the European Resuscitation Council on the need to call for assistance immediately is equal [5,6].

Experts from the European and Polish Resuscitation Council emphasize that the key elements of rescue operations are the ability to early assess the condition of the victim and to undertake resuscitation [7]. This area of activity was identified in literature as the first link in the survival chain [8]. Efficient performance of an examination determining the efficiency of critical systems leads to a reduction of complications resulting from tissue hypoxia, while also improving survival after cardiac arrest [9]. The assessment of basic life functions is a challenge even for experienced medical staff. Delaying resuscitation negatively affects the condition of the victim [10]. The assessment should be efficient and based on both breath detection and heart rate and be indicative to the symptoms. Performing a single element test does not confirm cardiac arrest [5,11].

In this research, rescuers working in teams of three proved to be more effective considering all the factors determining the quality of the victim’s assessment. The time spent on testing cardiovascular and respiratory performance, although not perfect, was closer to the pattern indicated in the ERC 2015 Guidelines than in the case of two-person teams. Teams of three-persons performed resuscitation activities noticeably faster. Perkins et al. confirm that the basis for making the assessment is care for proper and careful subsequent operations of the ABC algorithm (assessment of airway patency, breathing and circulation). The victim cannot be expected to breathe properly without previously cleared airways. Breathing assessment, lasting 3 s, will not be an objective indicator of respiratory performance. Assessment of the circulation, based only on monitor observation and ECG, is not enough to make further therapeutic decisions [12].

In this case, chest compression was implemented without undue delay. Due to the above, it can be stated that it is the triple units that are more effective in this aspect, and their work can more often lead to ROSC. Weissenberg et al. confirmed that survival outcomes depend on the time of beginning resuscitation, immediate chest compression by both accidental witnesses of the event and members of the emergency medical team. These factors can increase the prognosis for long-term survival of the victim two or even four times in particular cases [13].

Paramedics in three-person units far less frequently exceeded the indicated time limits. Care for minimizing interruptions was probably due to more staff who could devote time to implementing other procedures. Cheskes et al., often emphasize the legitimacy of minimizing breaks in chest pressure. They pay attention to maintaining compression time above 60% of the entire resuscitation cycle. The maximum pause should not exceed 10 s. Extending chest compression can have a fatal impact on the possibility of spontaneous circulation [14]. Stiel et al., Vadeboncoeur et al., Hostler et al., indicate an increase in resuscitation efficiency when chest compression depth is in the range of 4.5–5.5 cm [15,16,17]. Currently, the recommendations of international organizations such as ILCOR and ERC say that during resuscitation, chest compression should be sought to a depth of about 5, not exceeding 6 cm [18]. The analysis of this research results shows that both groups of teams did not reach the limit of 5 cm. The differences were relatively small, with a discreet advantage of three-person units. It might be assumed that in the above element the effectiveness of the tested units is comparable and needing improvement. Own research noted that there was a tendency to implement advanced techniques, intubation, vascular access, etc., at the expense of caring for good quality chest compression. Idris et al., in research results published in 2012 and 2015, indicate an increase in survival outcomes of patients with treated cardiac arrest in cases where a sternum pressure frequency of 100–120/min was used during therapy. The authors point out that frequency overstatement very often results in a decrease in the depth of compression and a significant increase in fatigue of paramedics, thus causing the effectiveness of therapy to decrease [19,20]. Analysis of the results of the study showed that both groups of teams maintained a frequency slightly exceeding 120 compressions/min. Double units turned out to be closer to the pattern. The second, immediately after chest compression, action undoubtedly affecting the effectiveness of resuscitation is proper oxygenation of the victim. Voss et al., in their publications set out the rules for conducting replacement breath. The fundamental action preceding the administration of air is the maintenance of airways, initially in an instrumental manner, at a later stage using specialized devices [21]. It was determined that the frequency of 10 breaths per minute, using a volume converter of 6–7 mL/kg (500–600 mL on average, which gives the effect of lifting the chest) completely protects the victim against the build-up of hypoxia [22]. The study shows that rescuers focus on pharmacotherapy, electrotherapy and other very absorbing techniques, while forgetting about the basis of resuscitation, namely high quality BLS. Such action is inappropriate, BLS was and will probably be the most important element of ALS.

Ventilation efficiency was another element under assessment. The results did not clearly indicate the advantage of a group of two or three. It turned out that the quality of the indicated procedure, like the level of chest compression, does not reach a satisfactory threshold. A small advantage of three-person units was observed, but this is not the level that could be expected from professional paramedics. In current standards, it is recommended to use medical oxygen from the beginning of therapy in such a way as to obtain its high concentration in the administered respiratory mixture as quickly as possible [5]. According to presented in the literature research results oxygen supplementation during ALS may lead to more frequent ROSC [22]. The element that distinguishes teams with more staffing is oxygen therapy. In this case, ventilation enriched with a respiratory mixture was used much more often. In view of the above, the advantage of the effectiveness of three-person teams should be recognized in this aspect.

During the verification of literature addressing the legitimacy of instrumental airway obstruction during ALS, positions were encountered calling into question the use of intubation and supraglottic methods. According to Fouche et al., there is not enough evidence for the positive impact of these procedures [23]. One can agree that this effective ventilation, not the introduction of an endotracheal tube, can lead to ROSC. In a situation where ventilation using a self-expanding bag is effective, immediate intubation should not be sought after. The optimal solution indicated by Voss et al., is the use of a combination of different techniques depending on the experience and skills of paramedics [21]. The anatomy of the injured party and the circumstances of conducting ALS should be assessed. The choice of instrumental airway obstruction technique should be based on all the variables indicated [5]. Soar et al. indicate that despite the lack of sufficient test results, the introduction of devices to facilitate the maintenance of airway patency can help in ventilation [5,7]. Analysis of the research material showed significant deficiencies in the ability to use the bag. Paramedics often use this equipment, but they do not care about its proper sealing to the victim’s face. This leads to an extension of the hypoxia period. It was observed that the teams, which as the first step decided to use supraglottic methods, obtained qualitatively good ventilation much faster. The visible advantage of alternative methods over intubation has been proven. The results of the study are confirmed in numerous publications discussing the high effectiveness of larynx tubes and I-gel masks. The advantage of alternative techniques is also the ability to conduct independent ventilation and chest compression (asynchronous technique). The condition that must be met is airway tightness [24]. Katte et al., Barr et al., Wiese et al., Gill et al., Sunde et al., Gahan et al., Schalk et al., indicate that after a short training, the ventilation efficiency obtained thanks to these devices reaches up to 80–100% [25,26,27,28,29,30]. During the examination, paramedics most often reached for I-gel masks. Wharton et al., Gatward et al., Duckett et al., indicate that even inexperienced doctors, nurses and paramedics are able to properly use this type of equipment [31,32,33], which is confirmed by the results of this research. Endotracheal intubation is a golden mean in maintaining airway patency. It protects the lower respiratory tract against aspiration of gastric content and allows asynchronous resuscitation [34]. It should be noted, however, that despite these advantages, significant paramedic experience is necessary for its efficient performance. It should only be used by qualified persons. During the observation of the research group, it was noted that paramedics deciding on rapid intubation usually obtained effective ventilation much later (regardless of the number of people in the team). The procedure took a great deal of time, introduced additional, unnecessary stress and chaos in action. Units that opted for simpler techniques and later intubated obtained advantage in the study. The stands of the authors of numerous publications confirm the results, three person teams do better than the ones that consist of only two members. Paramedics undertaking instrumental airway obstruction must faultlessly assess the location of the device and continuously monitor the quality of ventilation [5]. The most commonly used methods of verifying intubation quality include observing chest lifting symmetry, condensation, and the presence of respiratory murmurs over the pulmonary fields during ventilation. However, these are not the methods that give complete certainty. Kramer-Johansen et al., Lee et al., and Grmec indicate the unreliability of these techniques [34,35,36]. The use of capnography [36,37] is considered to be the optimal solution supporting the assessment of the position of the tube.

Perkins et al. draw attention to the issue of drug supply during treatment of cardiac arrest. We are increasingly finding opinions that question the legitimacy of Adrenaline application [12]. On the other hand, ERC’s resuscitation recommendations clearly indicate the need for adrenaline [7]. Due to the lack of sufficient evidence against the use of adrenaline, it is recommended to continue using the indicated preparation during advanced resuscitation procedures [38]. Similar data apply to amiodarone. There was no increase in survival outcomes until a person was discharged from hospital, but the effect of short-term improvement in condition was obtained [39]. It should be noted here that the drug delivery algorithm was divided depending on the mechanism of cardiac arrest. Defibrillation rhythms should be treated with adrenaline and amiodarone. The time of administration of the first doses of drugs determines the moment of the third defibrillation. If the discharge does not lead to ROSC, adrenaline, and amiodarone were introduced in the standard [40]. Currently recommended dosage of adrenaline limits the one-time application to 1 mg administered at 3–5-min intervals, and amiodarone up to 300 mg diluted in 5% glucose [5]. When treating non-defibrillation rhythms, we only use adrenaline in the algorithm. Three- and two-person units were verified for knowledge of recommendations regarding the limitation of the drug resource to two preparations, their dosage and application at recommended intervals. The intervals between doses often depended only on the case, no attention was paid to the passage of time. Fewer mistakes were noted during the dose assessment. Similar observations are indicated by Giberson et al. [41]. Noticeably fewer mistakes were made by paramedics in three-person teams.

Rapid rhythm analysis allows to effectively treat arrhythmias that require defibrillation. The chances of cardiac recovery with VF/VT treatment closely correlate with the passage of time and quality of BLS [42]. Blom et al. draw attention to the great importance of discharge in the first minutes of cardiac arrest [43]. An efficacy of 50–70% has been demonstrated for defibrillation performed within the first 3–5 min of diagnosis mechanism of cardiac cessation [44]. During the study, three-person teams gained an advantage in the electrotherapy procedure, obtaining shorter times until the first discharge. It should be mentioned, however, that the results obtained are not perfect. Another element tested is the ability to perform rhythm analysis at 2-min intervals. Recommendations on which the assessment basis is based are found in the ERC 2015 guidelines [5]. In this aspect, three-person teams again dominated. An analogy to the result of time control during use and pharmacovigilance is visible here. The data indicate that the activities are more effective when more personnel work at the scene.

The effectiveness of electrotherapy is also influenced by the minimization of breaks in bridge compression during camera preparation and the abolition of delay in the discharge decision in the event of recognition of defibrillation rhythms [5]. Up to 5–10 s break in BLS [45] may have a negative impact on the effectiveness of the discharge.

Energy grading is also an element that affects the quality of resuscitation while treating defibrillation rhythms. In the absence of a positive effect, it is recommended to gradually increase the power so that it reaches the maximum value at the third discharge [46]. In order to reduce resistance and improve the efficiency of defibrillation, it is suggested to use multifunctional electrodes [5] or to use a gel for defibrillation. Analyzing the quality of electrotherapy and referring to the opinions and tips of specialists, it was pointed out that knowledge of the principles of treatment of defibrillative arrhythmias is observably higher among teams of three.

To sum up all the elements affecting the quality of resuscitation, it can be clearly stated that the work of three paramedics is more efficient and definitely more effective. It was noted that in individual, isolated elements, the level of both groups is similar, however, in relation to the larger number of procedures imposed by the ERC, three-person teams prove to be more effective. According to the authors, the advantage of three-person teams over two-person teams in specific procedures used during cardiac arrest treatment may result from an additional person in the team. The number of procedures is the same in both cases; however, in a three-person team, the number of procedures is distributed among more personnel, thus, the speed and quality of their implementation increases.

## 5. Conclusions

Paramedics in three-person teams work more effectively, make a proper assessment of heart rhythm and monitor while undertaking advanced activities. The quality of ventilation and BLS in both groups studied is insufficient. Numerous errors have been observed in two-person teams over pharmacotherapy.

## Figures and Tables

**Table 1:** Comparison of the duration of two- and three-person basic emergency medical teams during advanced resuscitation procedures in adults under simulated conditions. Legend: (M—mean, SD—standard deviation, t—*t*-test value, *p*-value—probability value, Cl—confidence levels, dCohena—effect size).

Actions Taken in Accordance with the ALS Algorithm	Two-Person Team (*n* = 100)		Three-Person Team (*n* = 100)				99% Cl		
	M	SD	M	SD	t	*p*-Value	LL	UL	dCohena
Time to undertake rescue operations (s)	1.75	1.86	1.36	1.52	1.37	0.175	−0.17	0.94	0.23
Time to assess the condition of the victim (s)	7.46	5.51	9.76	4.68	−3.18	0.002	−3.73	−0.87	0.45
Time spent on the B/BC study (s)	2.92	4.17	4.33	3.61	−2.16	0.032	−2.71	−0.12	0.36
Time spent on C study (s)	5.25	3.11	7.26	2.01	−4.85	0.001	−2.83	−1.19	0.75
Time spent on a one-time BC study (s)	8.67	2.42	8.48	1.44	0.25	0.807	−1.38	1.76	0.11
Time to get the rhythm record (s)	1.14	1.19	0.52	0.52	4.81	0.001	0.37	0.88	0.68
Time of first defibrillation (s)	1.28	1.06	0.56	0.6	4.18	0.001	0.38	1.06	0.84
Time to undertake effective chest compression(s)	0.29	0.24	0.22	0.1	2.77	0.007	0.02	0.13	0.39
Frequency of chest compressions per minute	125	17.1	127	14.71	−1.15	0.253	−7.04	1.86	0,16
The quality of the parameters of chest compressions	160.42	42.6	131	37.76	5.14	0.001	18.01	40.45	0.73
Time to obtain effective ventilation with a self-expanding bag (s)	1.91	6.9	0.43	0.25	0.98	0.34	−1.67	4.62	0.41
Average ventilation volume (mL)	442	86	457	78.76	−1.3	0.196	−38.14	7.86	0.18
Average depth chest compression (mm)	45.4	8.04	48	6.04	−2.65	0.009	−4.64	−0.68	0.37
Time to achieve proper ventilation using percortation methods (s)	1.67	1.08	1.12	0.55	4.08	0.001	0.28	0.82	0.63
Time to achieve proper ventilation by intubation (s)	3.38	1.73	3.11	2.35	0.47	0.643	−0.89	1.43	0.13
Number of tests after which endotracheal intubation was obtained	1.3	0.54	1.18	0.46	0.9	0.37	−0.14	0.36	0.23
Time of first dose of Adrenaline (s)	2.56	1.03	1.79	0.77	4.24	0.001	0.41	1.13	0.85
Time after which additional help was called (s)	278	148	194	140.73	3.92	0.001	41.68	126.3	0.58

## Data Availability

Datasets used and analyzed during the current study are available from the corresponding author on reasonable rquest.

## Supplementary Materials

The following are available online at https://www.mdpi.com/article/10.3390/ijerph18094834/s1, Supplementary S1: Simulation Evaluation Checklist.
