# Supplementary material for: Comparative Analysis of the Effectiveness of Performing Advanced Resuscitation Procedures Undertaken by Two- and Three- Person Basic Medical Rescue Teams in Adults under Simulated Conditions
Source: Int J Environ Res Public Health. 2021 Apr 30;18(9):4834. doi: 10.3390/ijerph18094834 (PMC8124675; doi:10.3390/ijerph18094834)
Supplement: Supplementary file 1 [file ijerph-18-04834-s001.zip › ijerph-1164494-supplementary.pdf]

1 PRIMARY ASSESSMENT

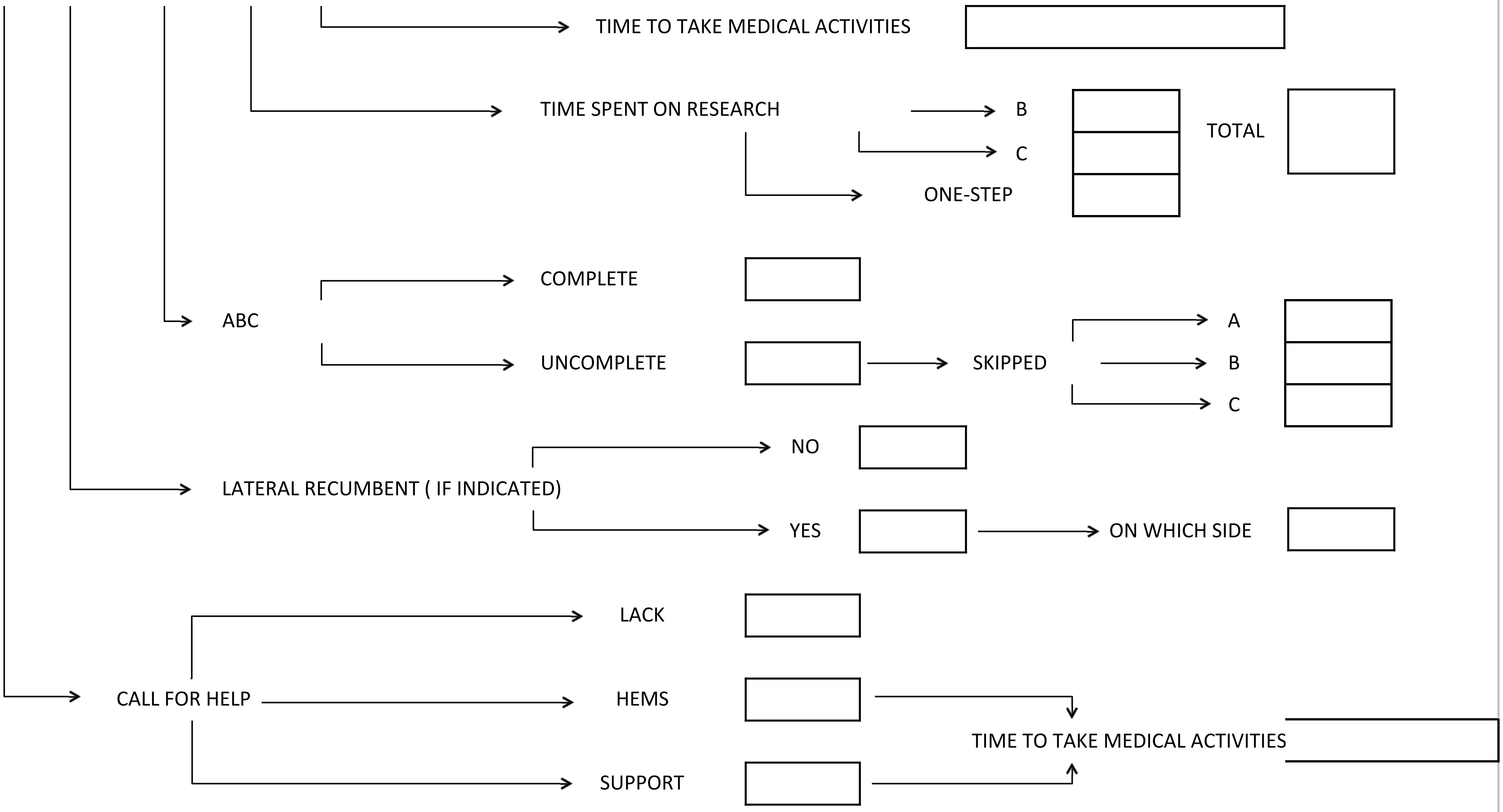

2 BLS

- TIME OF IDENTIFIED CARDIAC ARREST
  - B
  - C
- BEGINNING OF CHEST COMPRESSIO(IF INDICATED)
  - YES
  - N
- BEGINNING OF VENTILATION (IF INDICATED)
  - YES → QUANTITY
  - NO
- TECHNIQUE OF CHEST COMPRESSION
  - YES
  - NO
- QUALITY ACCORDING TO THE ANALYSIS OF A COMPUTER PRINT ATTACHMENT

3 OXYGEN THERAPY

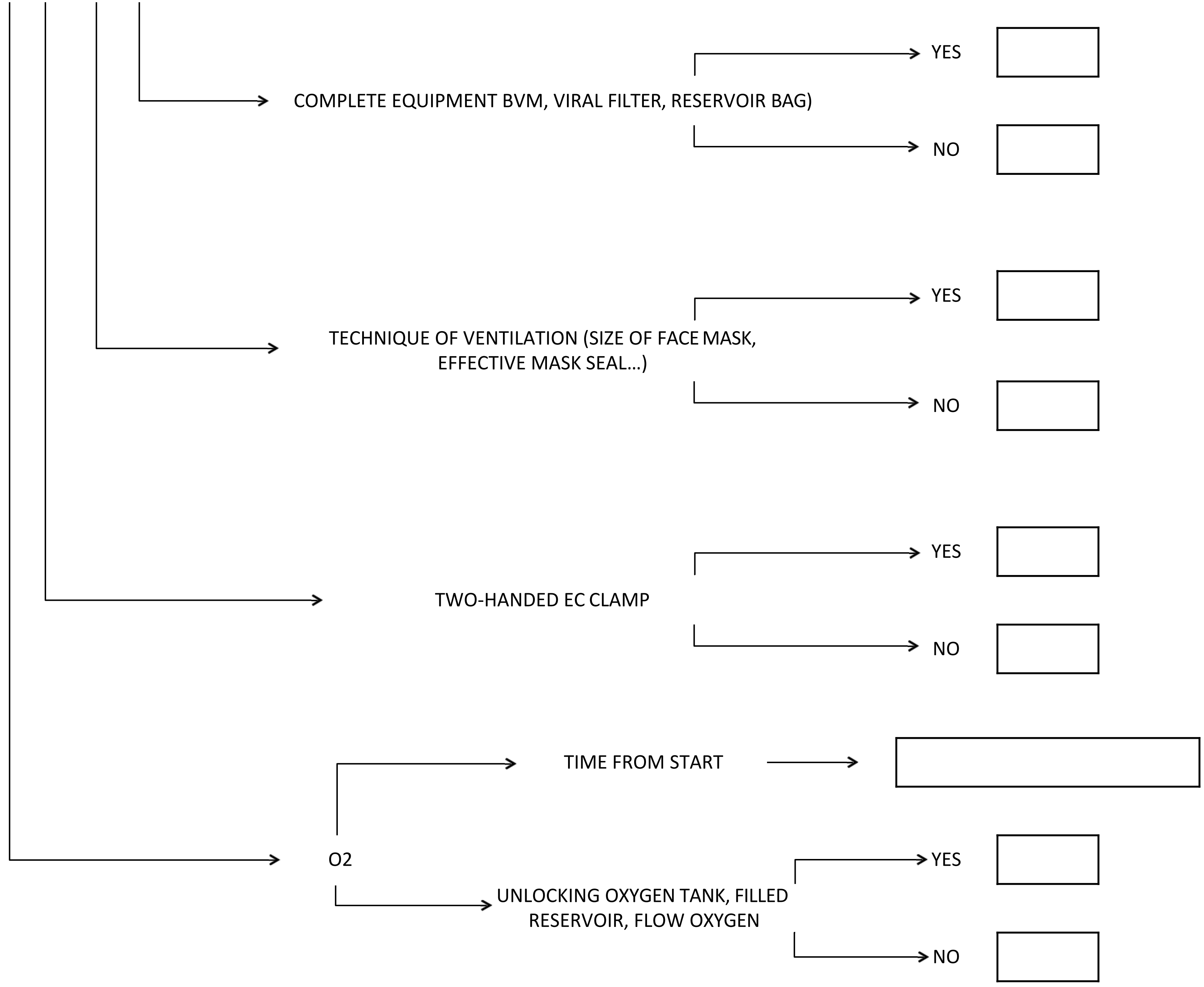

AIRWAYS MANAGEMENT- A

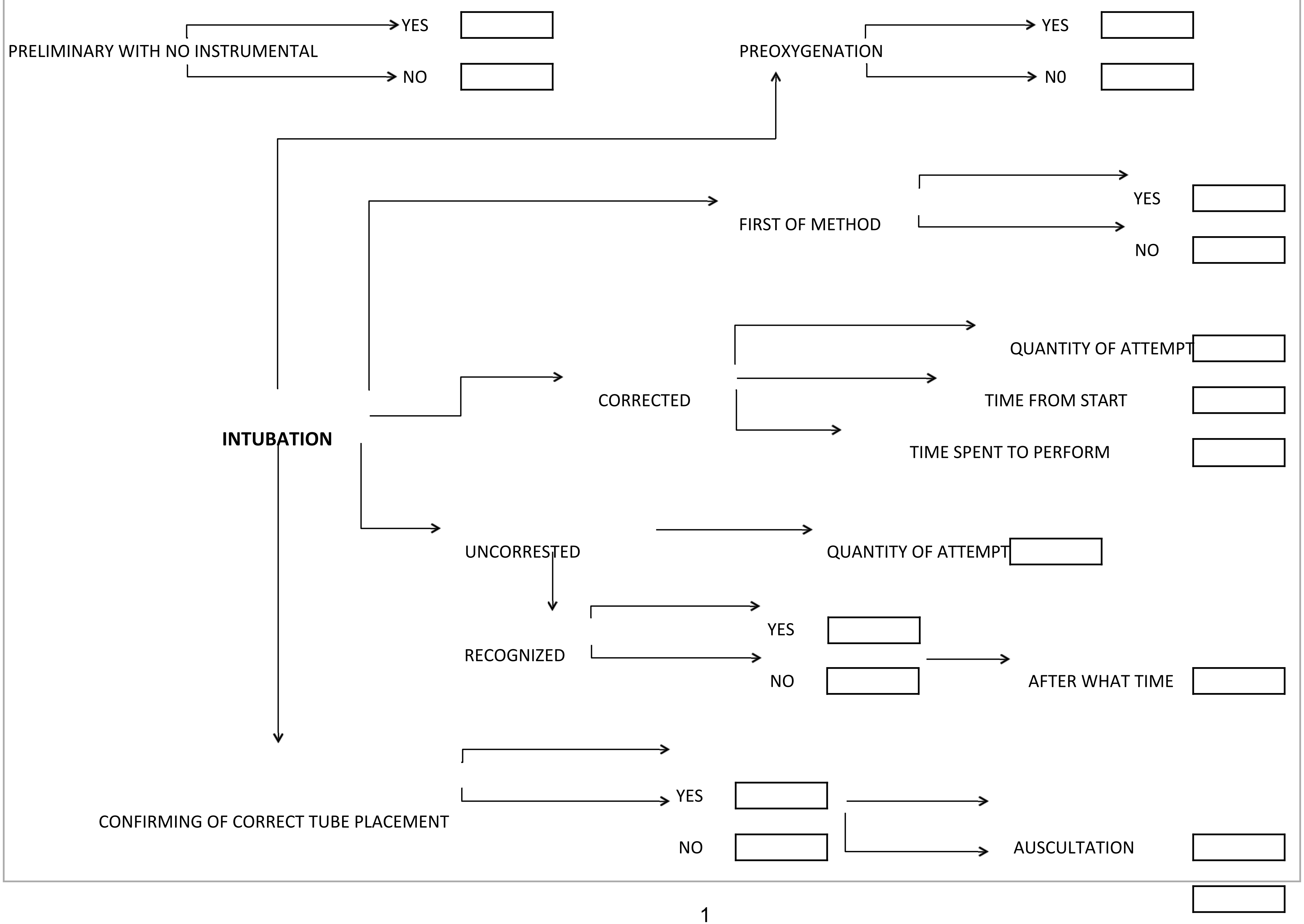

CAPNOGRA

PHY

AIRWAYS MANAGEMENT - B

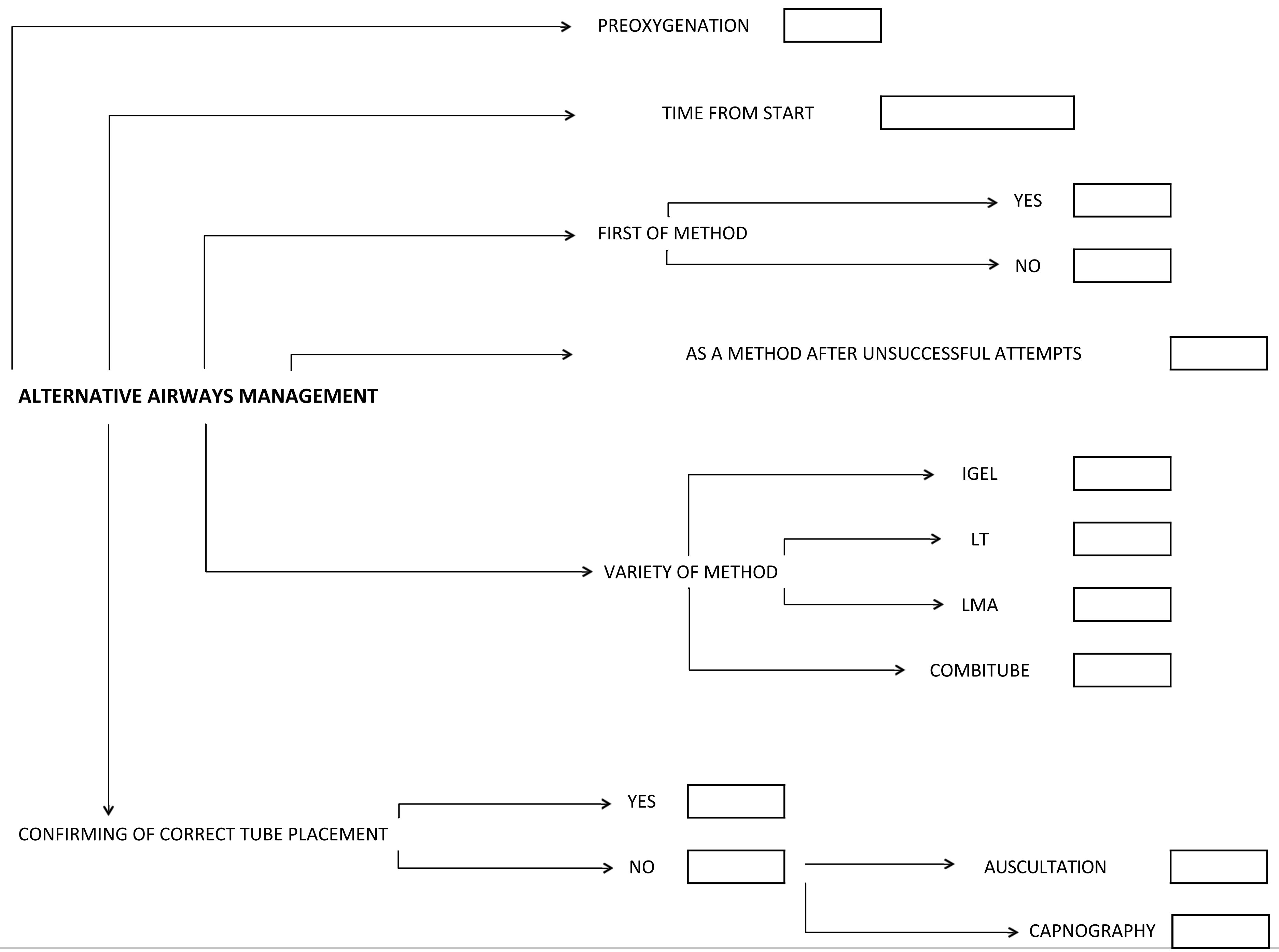

4 MONITORING AND ELECTROTHERAPY

QUICK LOOK

ELECTRODES

TIME FROM START

VF

CORRECTED

DIAGNOSIS

VT

IMMEDIATELY DEFIBRILLATION

DECISION OF DEFIBRILLATION

DELAYED DEFIBRILLATION

TIME

AYS

UNCORRECTED

PEA

YES

REPEATED RESEARCH OF C IN VT (BEFORE DEFIBRILLATION)

NO

DISCHARGE OF DEFIBRILLATION 1 2 3 4

YES

DEFIBRILLATION EVERY 2-MINUTES

N0

ANOTHER TIME

YES

NALYSIS EVERY 2-MINUT

NO

CO ILE

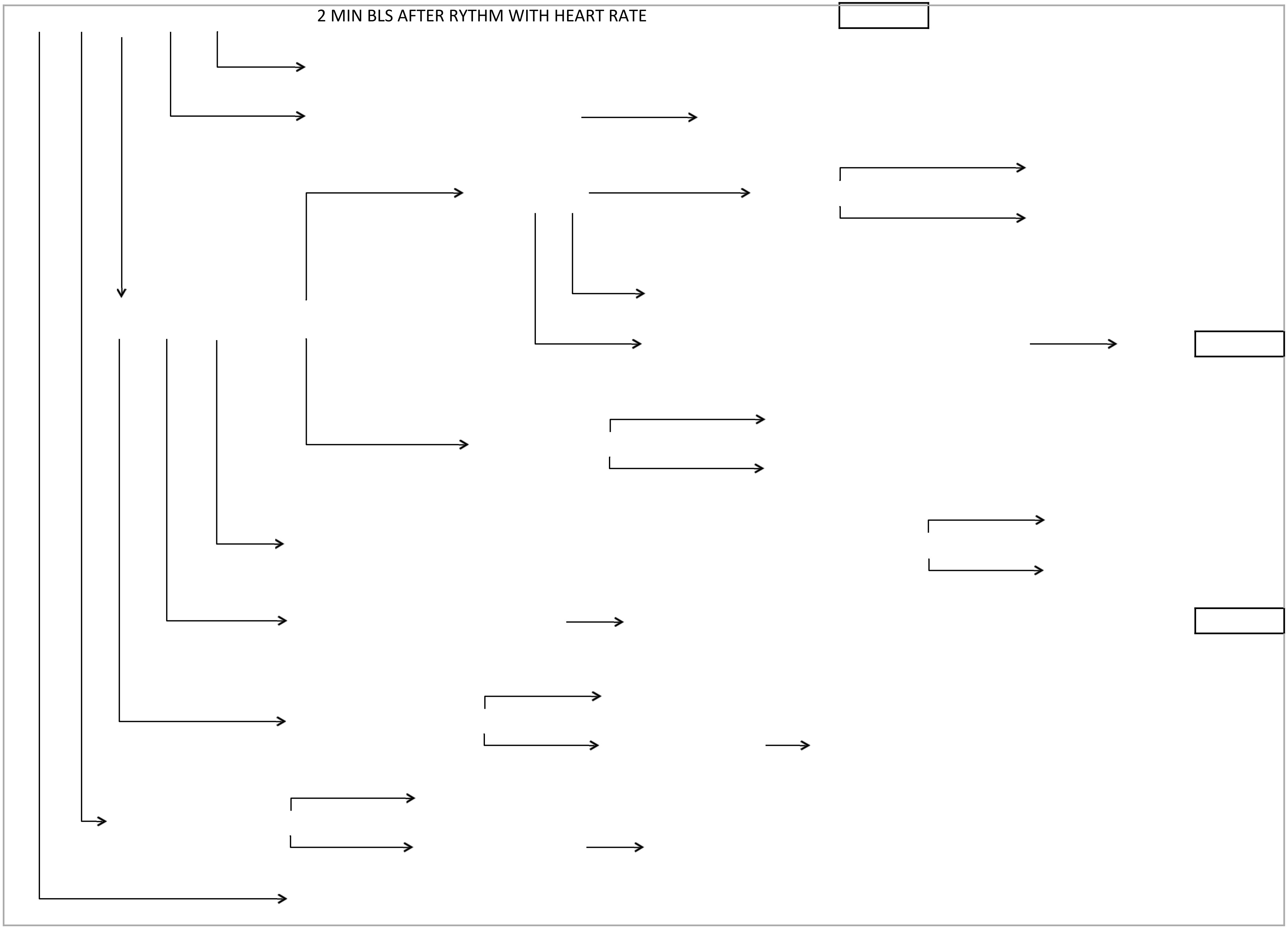
2 MIN BLS AFTER RYTHM WITH HEART RATE

5 PHARMACOLOGICAL TREATMENT

OBTAINING VASCULAR ACCESS

INTRAVENOUS [ ] TIME [ ]

INTRAOSSEOUS [ ] TIME [ ]

NUMBER OF ACCESSES USED [ ]

ANOTHER DRUG DELIVERY SYSTEM

HOW [ ]

INDICATIONS [ ]

RECTITUDE OF ALGORITHM

SHOCKABLE RHYTHMS

YES [ ]

NO [ ]

NON-SHOCKABLE RHYTHMS

YES [ ]

NO [ ]

INTRAVENOUS FLUID THERAPY

NO [ ]

YES [ ]

TYPE OF MEDICAL PREPARATION [ ]

QUANTITY [ ]

SZYBKOŚC PODANIA [ ]

MEDICAMENTS

NO [ ]

YES [ ]

NAME	TIME
